# Genomic Characterization of *Listeria monocytogenes* Isolates Associated with Clinical Listeriosis and the Food Production Environment in Ireland

**DOI:** 10.3390/genes9030171

**Published:** 2018-03-20

**Authors:** Amber Hilliard, Dara Leong, Amy O’Callaghan, Eamonn P. Culligan, Ciara A. Morgan, Niall DeLappe, Colin Hill, Kieran Jordan, Martin Cormican, Cormac G.M. Gahan

**Affiliations:** 1 APC Microbiome Ireland, University College Cork, Cork T12 K8AF, Ireland; amber.hilliard@ucc.ie (A.H.); ocallaghanamy86@gmail.com (A.O.); eamonn.culligan@cit.ie (E.P.C.); ciara.morgan@umail.ucc.ie (C.A.M.); c.hill@ucc.ie (C.H.); 2 School of Microbiology, University College Cork, Cork T12 K8AF, Ireland; 3 Teagasc Food Research Centre, Moorepark, Fermoy, Cork P61 C996, Ireland; dara.leong@teagasc.ie (D.L.); kieran.jordan@teagasc.ie (K.J.); 4 National Salmonella, Shigella and Listeria Reference Laboratory, Galway SW4 671, Ireland; niall.delappe@hse.ie; 5 School of Medicine, National University of Ireland, Galway H91 TK33, Ireland; martin.cormican@hse.ie; 6 School of Pharmacy, University College Cork, Cork T12 K8AF, Ireland

**Keywords:** *Listeria monocytogenes*, clinical, genome, genomics, sequence, single nucleotide polymorphism, SNP

## Abstract

*Listeria monocytogenes* is a major human foodborne pathogen that is prevalent in the natural environment and has a high case fatality rate. Whole genome sequencing (WGS) analysis has emerged as a valuable methodology for the classification of *L. monocytogenes* isolates and the identification of virulence islands that may influence infectivity. In this study, WGS was used to provide an insight into 25 *L. monocytogenes* isolates from cases of clinical infection in Ireland between 2013 and 2015. Clinical strains were either lineage I (14 isolates) or lineage II (11 isolates), with 12 clonal complexes (CC) represented, of which CC1 (6) and CC101 (4) were the most common. Single nucleotide polymorphism (SNP) analysis demonstrated that clinical isolates from mother–infant pairs (one isolate from the mother and one from the infant) were highly related (3 SNP differences in each) and also identified close similarities between isolates from otherwise distinct cases (1 SNP difference). Clinical strains were positive for common virulence-associated loci and 13 isolates harbour the LIPI-3 locus. Pulsed-field gel electrophoresis (PFGE) was used to compare strains to a database of 1300 Irish food and food processing environment isolates and determined that 64% of clinical pulsotypes were previously encountered in the food or food processing environment. Five of the matching food and food processing environment isolates were sequenced and results demonstrated a correlation between pulsotype and genotype. Overall, the work provides insights into the nature of *L. monocytogenes* strains currently causing clinical disease in Ireland and indicates that similar isolates can be found in the food or food processing environment.

## 1. Introduction

*Listeria monocytogenes* is the causative agent of listeriosis, a serious infection that can manifest as meningitis and/or septicaemia in adults, infection of the fetus and miscarriage in pregnant women, or neonatal infection [1,2]. Although the disease is relatively rare, listeriosis is severe, with high hospitalisation and mortality rates [1,3]. In 2014, there were 2161 confirmed cases of human listeriosis in the European Union (EU) [4]. The fatality rate among 1524 confirmed cases with known outcome was 17.7% (270 cases), indicating the potential for this pathogen to pose a significant public health concern. In Ireland, listeriosis is a notifiable disease and the number of reported cases has been subject to an increase recently from eight cases in 2013, 15 cases in 2014 to 19 cases in 2015. The case incidence rate for 2015 was approximately 0.41 per 100,000 population, which is below the EU average of 0.48 cases per 100,000 for the same year [4,5]. However, listeriosis in Ireland remains a significant hazard for immunocompromised persons and other vulnerable groups, especially the elderly. A recent report suggests a trend towards an increased percentage of cases in adults (in particular adults over 65 years of age), which present as blood stream infection or meningitis [5].

*L. monocytogenes* consists of four evolutionary lineages and 13 serotypes with serotypes 4b and 1/2b (in lineage I) and 1/2a and 1/2c (in lineage II) being the most common causes of human listeriosis [6]. Whole genome sequencing (WGS) and subsequent genomic analyses can differentiate isolates that are otherwise indistinguishable by other typing methodologies (including pulsed-field gel electrophoresis (PFGE)) and can also produce significant insights into loci associated with pathogenesis in virulent or hypervirulent strains [7,8]. WGS and multi-locus sequence typing (MLST) can subdivide isolates according to sequence type (ST) or clonal complex (CC) whilst analysis of single nucleotide polymorphisms (SNPs) provides even greater granularity for the purposes of strain comparisons in the context of molecular epidemiological investigations [9,10].

Crucial to *L. monocytogenes* pathogenesis is an ability to invade host cells using internalins [11]. In particular, the interaction between internalin A (InlA) and host E-cadherin is essential for oral infection [12,13]. However, a significant proportion of environmental and food isolates of *L. monocytogenes* produce a truncated form of the InlA protein due to premature stop codons in the *inlA* gene and are therefore significantly compromised in virulence potential [14,15]. *L. monocytogenes* also encode genomic islands, known as *Listeria* pathogenicity islands (LIPIs), which play important roles in the virulence of the pathogen. LIPI-1 is the major pathogenicity island (encoding factors that are essential for phagosomal escape (listeriolysin O (LLO)) and cell-to-cell spread (ActA)) and is well conserved across strains independent of lineage [16]. The presence of LIPI-3, which includes a second haemolysin known as listeriolysin S (LLS), is strongly associated with lineage I strains, including a number of serotype 4b strains that have caused epidemic listeriosis [17]. Recent work suggests that LLS functions as a bacteriocin and plays a significant role in the gastrointestinal phase of *Listeria* infection [18]. Finally, LIPI-4, which encodes a cellobiose family phosphotransferase (PTS) system, is strongly associated with certain lineage I strains that are associated with invasion of the central nervous system (CNS) [7]. In turn, other factors may play a role in the environmental survival of specific isolates [19]. Such loci include a five gene cluster known as the stress survival islet 1 (SSI-1), which may contribute to the survival of cells in suboptimal conditions including high salt and low pH [20].

The objectives of this study were to use WGS to characterise distribution of virulence determinants in Irish clinical *L. monocytogenes* isolates, to compare methods for determining relatedness of the isolates (PFGE, ST, CC, core genome and SNP analysis) and to relate clinical isolates and food-related isolates. The data reveal the predominant sequence types causing clinical disease in Ireland, highlight the presence or absence of particular virulence associated genes and indicate that similar strains are present in foods and food processing environments.

## 2. Materials and Methods

### 2.1. Bacterial Isolates

To determine the genetic diversity and to identify the genetic characteristics of *L. monocytogenes*, a total of 25 Irish *L. monocytogenes* isolates from clinical samples were obtained from the national *Listeria monocytogenes* collection database at the National Reference Laboratory Service, University Hospital Galway (Table 1). These strains have also been included in a pan EU study of similarity between *L. monocytogenes* isolates by cgMLST. In addition, the PFGE profiles of the 25 clinical isolates were compared to a database of about 1300 isolates obtained from about 70 small food processing companies from 2013 to 2015 [21]. Six strains of *L. monocytogenes* from food and the food processing environment (five with similar PFGE profiles to the clinical isolates and one unrelated control strain), were sequenced to investigate whether there is genomic relatedness between food/food environment isolates and the clinical cases (Table 1). A subset of suitable reference strains were also selected from publicly available complete genomes including EGD-e (NC_003210.1), F2365 (NC_002973.6) and CLIP 80459 (NC_012488.1).

### 2.2. Pulsed Field Gel Electrophoresis

Pulsed field gel electrophoresis analysis was carried out using the International Standard PulseNet 2013 protocol. The DNA was digested with 10 U/µL of the restriction enzyme Sgs1 (Asc1) FastDigest (Fisher Scientific, Dublin, Ireland) and 50 U/µL of the restriction enzyme ApaI FastDigest (Fisher Scientific); the restricted DNA was run in a 1% SeaKem Gold agarose gel for 21 h as described in the PulseNet protocol, on a CHEF-DR III (Bio-Rad, Hercules, CA, USA). After staining with 1 µg/mL ethidium bromide solution, the gels were observed with the Alpha Imager (Alpha Innotech, Kasendorf, Germany). Analysis of the gels was performed with BioNumerics v7.0 software (Applied Maths, Sint-Martens-Latem, Belgium) using UPGMA (unweighted pair group method with averages) and the Pearson coefficient with 1% tolerance.

### 2.3. Genome Sequencing and Annotation

The 25 clinical isolates were sequenced previously by our group [22]. The same protocol and platform was utilised to sequence the 6 selected food/food environment isolates described herein. Briefly, DNA was prepared using the GenElute Bacterial Genomic DNA kit (Sigma Aldrich, St. Louis, MO, USA) as per the manufacturer’s instructions. Library preparation and 250-bp paired end sequencing were performed using the Illumina HiSeq 2500 platform (Microbes NG, University of Birmingham, Birmingham, UK). Raw reads were mapped to a reference genome using the Burrows-Wheeler aligner-maximum exact matches (BWA-mem) [23] and de novo assembly was performed using the SPAdes genome assembler [24]. Contigs were re-ordered using Mauve aligner (v2.4.0) [25]. Prediction of putative open reading frames (ORFs) was performed using PRODIGAL prediction software [26] and supported by BLASTX and BLASTP [27]. Artemis [28] was employed for visualisation and manual editing in order to verify, and, where necessary, redefine the start of predicted coding regions. Genomes have been submitted to GenBank and assigned the following accession numbers (in brackets); L970 (PJJD00000000), L1445 (PJJG00000000), L1976 (PJJI00000000), L2113 (PJJE00000000), L2256 (PJJH00000000), L2259 (PJJF00000000).

### 2.4. Multilocus Sequence Typing and Clonal Complex

In order to determine the level of phylogenetic diversity between isolates, STs were determined by MLST using seven housekeeping genes, including ABC transporter *abcZ*, beta-glucosidase *bglA*, catalase *cat*, succinyl diaminopimelate dessucinylase *dapE*, D-amino acid aminotransferase *dat*, L-lactate dehydrogenase *ldh* and histidine kinase *IhkA*. The contig files for each of the draft genomes were uploaded to the Center for Genomic Epidemiology MLST 1.8 with *L. monocytogenes* as the MLST scheme. The clonal complex (CC) was defined based on the MLST profile of the isolate having matching profiles at 6 out of 7 genes [8,29]. The evolutionary history was inferred by using the Maximum Likelihood method based on the Tamura 3-parameter model [30]. The tree with the highest log likelihood is shown. The percentage of trees in which the associated taxa clustered together is shown next to the branches. Initial tree(s) for the heuristic search were obtained automatically by applying Neighbor-Join and BioNJ algorithms to a matrix of pairwise distances estimated using the Maximum Composite Likelihood (MCL) approach, and then selecting the topology with superior log likelihood value. A discrete γ distribution was used to model evolutionary rate differences among sites (5 categories (+*G*, parameter = 0.5970)). The rate variation model allowed for some sites to be evolutionarily invariable ([+*I*], 77.4196% sites). The tree is drawn to scale, with branch lengths measured in the number of substitutions per site. The analysis involved 31 nucleotide sequences. Codon positions included were 1st + 2nd + 3rd + Noncoding. All positions with less than 95% site coverage were eliminated. That is, fewer than 5% alignment gaps, missing data, and ambiguous bases were allowed at any position. There were a total of 3288 positions in the final dataset. Evolutionary analyses were conducted in MEGA7 [31].

### 2.5. Single Nucleotide Polymorphisms Analysis

Single nucleotide polymorphisms were identified using the CSI Phylogeny 1.4 pipeline available on the Center for Genomic Epidemiology [32]. Separate analyses were carried out on 4b serotype strains (with strain F2365 as a reference) and 1/2a serotype strains (with EGDe as a reference). Phylogenetic trees were generated using the CSI Phylogeny tool available at the Center of Genomic Epidemiology. Multi-FASTA files, containing the sequences for each contig of the draft genome, were uploaded to the CSI Phylogeny 1.4 pipeline. Default parameters were used [32]. A maximum likelihood tree was created using FastTree and the subsequent Newick file was visualised using Figtree v1.4.2 [33]. The evolutionary history was inferred by using the Maximum Likelihood method, based on the General Time Reversible model [34]. The tree with the highest log likelihood is shown. The percentage of trees in which the associated taxa clustered together is shown next to the branches. Initial tree(s) for the heuristic search were obtained automatically by applying Neighbor-Join and BioNJ algorithms to a matrix of pairwise distances estimated using the MCL approach, and then selecting the topology with superior log likelihood value. The trees are drawn to scale, with branch lengths measured in the number of substitutions per site. Codon positions included were 1st + 2nd + 3rd + Noncoding. All positions with less than 95% site coverage were eliminated. That is, fewer than 5% alignment gaps, missing data, and ambiguous bases were allowed at any position. Evolutionary analyses were conducted in MEGA7 [31].

### 2.6. Public Data Sources

Several publicly available genome sequences of various MLST types of *L. monocytogenes* were included in the SNP analysis. These were complete or draft sequences of the *L. monocytogenes* genome (Supplementary Material File S1). The genome sequences were downloaded from the Genbank database.

### 2.7. Pan- and Core-Genomic Profiling of Protein-Coding Genes

A comparative analysis of the genome content using GView [35] generated a pangenome profile of the *L. monocytogenes* isolates. The pangenome was constructed by iteratively appending unique regions onto an initial seed genome. Genbank files for three reference strains (F2365 seed genome, Clip80459 and EGD-e) were compared with the clinical and food/food processing environment isolates.

PanCoreGen [36] was subsequently used to construct the pan-genomic database of orthogolous genes using the clinical and food associated isolates along with suitable references including F2365, CLIP80459 and EGD-e. This algorithm performs BLASTN against genes from each annotated genome to detect orthologs in the query genomes based on user-defined threshold values of nucleotide identity and length-coverage. Stringent cut-off values (80–95%) for both sequence-identity and gene length-coverage were used to determine the set of core genes. A complete profile of core, mosaic and strain-specific genes was created which was processed to generate a matrix of 1s and 0s to indicate the presence or absence of genes across each genome. The pan-genome distance-matrix was generated on the basis of distance between profiles using the binary distance measure.

## 3. Results and Discussion

### 3.1. Serotypes and Clonal Complexes of Clinical Isolates

The objectives of this study were to use WGS to characterise virulence determinants of Irish clinical *L. monocytogenes* isolates, in order to compare a variety of methods for determining relatedness of the isolates (PFGE, ST, CC, core genome and SNP analysis) and to relate clinical isolates to a selection of food/food processing environment isolates from a three year longitudinal study [21]. Twenty-five isolates were available from the National Reference Laboratory Service culture collection and as expected strains were either lineage 1 (serotype 4b) or lineage II (serotype 1/2a or 1/2c). Following WGS [22], the clinical isolates were also analysed for ST using the Center for Genomic Epidemiology MLST tool (Table 1). The isolates included 13 sequence types which corresponded to twelve CCs [8,29]. Clonal complex 1 (CC1) accounts for 7/25 isolates from sampled Irish clinical cases. Other predominant clones include CC101 (4/25), CC121 (3/25) and CC6 (3/25).

Data indicates that the number of reported listeriosis infections in the EU has increased by 17% since 2013 and that serogroup 4 has become the most prevalent serogroup (EFSA, 2015). Similarly, Ireland has experienced an increase in cases of listeriosis since 2013 (HPSC, 2015) with serotype 4b the most prevalent serotype followed by 1/2a. The majority of CC identified in our cohort of isolates were CC1, CC2, CC6 and CC101, which are predominantly associated with clinical sources [7], while CC121 is primarily considered a food-associated isolate [7,37]. A single isolate (from a cerebrospinal fluid (CSF) sample) was identified as belonging to CC4, a clonal complex that is generally prevalent in cases of human listeriosis and is neuroinvasive and hypervirulent [7]. Most CCs are stable over decades, however, CC101, which was common in the 1950s but decreased subsequently, has begun to re-emerge globally [37]. It is also worth noting that ST-1/CC1 accounts for approximately 30% of the isolates in this study. This clone is typically associated with clinical sources [7] and has the same ST as several outbreak strains including an isolate (F2365) that was responsible for epidemic listeriosis associated with a Mexican-style cheese in 1985 [38].

### 3.2. Relationship between Sequence Type and PFGE Profiles

Pulse-field gel electrophoresis analysis of the 25 clinical *L. monocytogenes* isolates was undertaken in order to allow comparisons with a database of *L. monocytogenes* strains isolated from ready-to-eat (RTE) foods and food production facilities in Ireland [21]. Pulsotype numbers were assigned using both enzymes, based upon an in-house classification system that was previously used to type food and food processing environment isolates [21,39]. The data indicate (Table 1) that certain clinical isolates dating from 2013–2015 closely matched strains that were isolated from contaminated foods and food processing environments in Ireland during the same time period. In particular, pulsotypes P2, P6, P31 and P59 were commonly associated with RTE foods or food processing environments across multiple food sectors and were considered as persistent strains within food production facilities and associated foods (isolated more than once from the same facility over 6 months apart) [21]. Interestingly, the clinical isolates MQ140030 (CC4), MQ150011 (CC20) and MQ140032 (CC90) represented pulsotypes that were not previously encountered in our previous analysis of Irish food and environmental strains [21] and most likely represent isolates that are very rare in foods and food environments in Ireland.

To allow further comparisons, six food-associated strains were sequenced, five of which (strains L2259, L970, L2113, L2256, L1445) have pulsotypes that match those of the clinical isolates (one strain, L1976, was used as a control outlier for phylogenetic comparisons). In this, admittedly low, sample set, pulsotype generally correlated with CC as defined by MLST, as expected. For instance, the pulsotype P2 isolates L2113 and L970 which were isolated from a food processing environment, were both CC1 as determined following WGS, and closely related to clinical CC1 strains.

**Figure 1:**
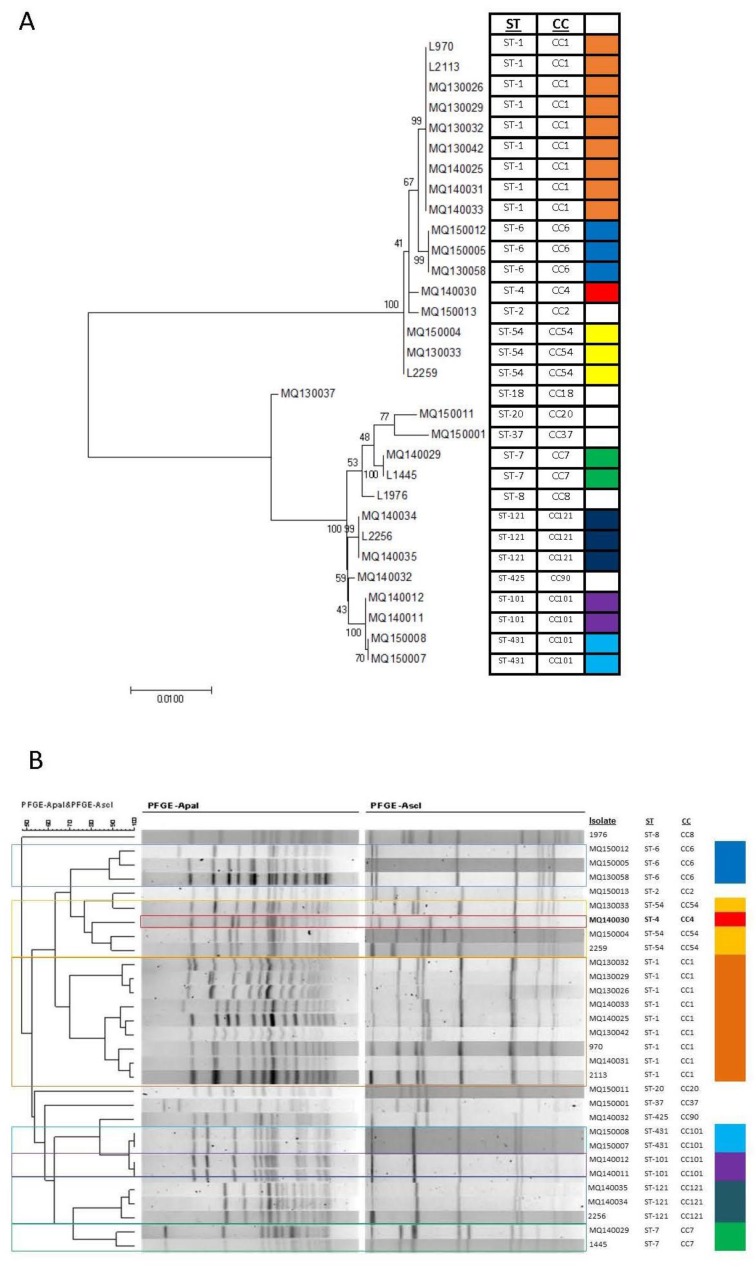
(**A**) Clustering of isolates based upon multi-locus sequence typing (MLST). The evolutionary history was inferred by using the Maximum Likelihood method based on the Tamura 3-parameter model [30] as outlined in detail in Materials and Methods. The tree with the highest log likelihood (−5971.3729) is shown. The percentage of trees in which the associated taxa clustered together is shown next to the branches. The tree is drawn to scale, with branch lengths measured in the number of substitutions per site. Evolutionary analyses were conducted in MEGA7 [31]; (**B**) Clustering of isolates based upon pulsed field gel electrophoresis (PFGE). Dendograms were generated with BioNumerics v7.0 software (Applied Maths) using UPGMA (unweighted pair group method with averages) and the Pearson coefficient with 1% tolerance. Using either method (MLST or PFGE) strains cluster into two distinct groups dependent upon lineage. ST: sequence type; CC: clonal complex.

Figure 1A shows a maximum likelihood phylogeny constructed from the nucleotide sequences of the seven house-keeping genes used for sequence typing of *L. monocytogenes* by MLST while Figure 1B shows a dendrogram of the PFGE profiles. Both techniques are used to sub-type isolates, and can give results that are not comparable. Using MLST, the three CC54 isolates (two clinical and one food isolate) are shown as indistinguishable, while the ST-4 isolate MQ140030 is closely related. Using PFGE, the three CC54 isolates are more or less distinguishable, particularly MQ130033 and MQ150004, which show <80% similarity, while L2259 and MQ150004 show about 90% similarity. The ST-4 isolate shows about 80% similarity to the ST-54 isolates. On the other hand, strains MQ150013 and MQ130033 are indistinguishable (>90% similarity) by PFGE, but can be differentiated by ST.

Although PFGE has greater discriminatory power than MLST, it does not have sufficient discriminatory power to consistently distinguish epidemiologically unrelated strains of *L. monocytogenes* [6]. In addition, while the methods of analysis have been standardised internationally, the naming of profiles unfortunately has not. This makes the comparison of strains amongst research groups and epidemiological monitoring bodies difficult. MLST based on the sequence of seven housekeeping genes overcomes some of these limitations and provides a highly standardised genotype that allows comparison with strains isolated internationally [7].

### 3.3. Single-Nucleotide Polymorphism Analysis of Isolates

**Figure 2:**
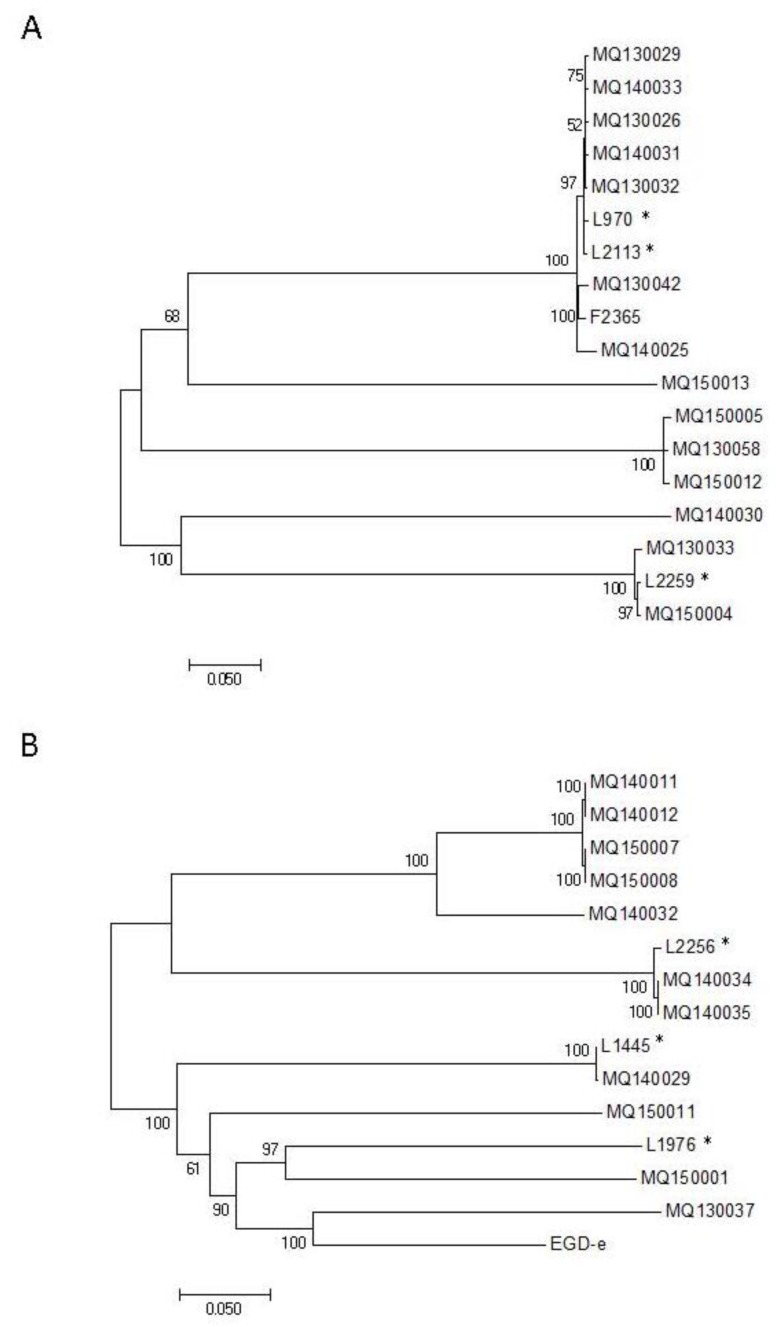
Phylogeny of the isolates as determined by single nucleotide polymorphism (SNP) analysis. (**A**) All serotype 4b isolates were compared with strain F2365 as the reference genome. The evolutionary history was inferred by using the Maximum Likelihood method based on the General Time Reversible model [34]. The tree with the highest log likelihood (−67933.7374) is shown. The percentage of trees in which the associated taxa clustered together is shown next to the branches. Initial tree(s) for the heuristic search were obtained automatically by applying Neighbor-Join and BioNJ algorithms to a matrix of pairwise distances estimated using the Maximum Composite Likelihood (MCL) approach, and then selecting the topology with superior log likelihood value. The tree is drawn to scale, with branch lengths measured in the number of substitutions per site. The analysis involved 18 nucleotide sequences. Codon positions included were 1st + 2nd + 3rd + Noncoding. All positions with less than 95% site coverage were eliminated. That is, fewer than 5% alignment gaps, missing data, and ambiguous bases were allowed at any position. There were a total of 12,069 positions in the final dataset. Evolutionary analyses were conducted in MEGA7 [31]. (**B**) All serotype 1/2a isolates were compared with strain EGDe as the reference genome. The evolutionary history was inferred by using the Maximum Likelihood method based on the General Time Reversible model [34]. The tree with the highest log likelihood (−214301.5165) is shown. The tree is drawn to scale, with branch lengths measured in the number of substitutions per site. The analysis involved 15 nucleotide sequences. Codon positions included were 1st + 2nd + 3rd + Noncoding. All positions with less than 95% site coverage were eliminated. That is, fewer than 5% alignment gaps, missing data, and ambiguous bases were allowed at any position. There were a total of 30,102 positions in the final dataset. Evolutionary analyses were conducted in MEGA7 [31]. * Indicates strains from a food production source.

Due to the significant number of ST-1 isolates in both the clinical (28%) and food groups (33%), SNP analysis was carried out to assess whether any of the isolates may be closely linked. To obtain a broad overview of the SNP profiles, each isolate was initially compared to a reference strain of the same serotype. All serotype 4b isolates were initially compared to the reference F2365 (Supplementary Table S1) and all serotype 1/2a isolates were compared to the reference EGDe (Supplementary Table S2). The serotype 4b isolates differed by between a minimum of 41 and a maximum of 5862 SNPs while the serotype 1/2a isolates differed by a minimum of 3 and a maximum of 12,515 SNPs. A maximum likelihood tree was generated for each comparison showing the relationship between the isolates. The SNP tree clustered the *L. monocytogenes* isolates according to their ST/CC (Figure 2).

For reference-based methods of SNP analysis, the choice of reference genome can significantly influence the results [40]. Comparisons of genetically distant groups of isolates using a single reference may result in loss of resolution particularly if the chosen reference is genetically distant from the isolates under investigation [41]. Following the initial analyses, each cluster of closely related isolates (i.e., a single ST or CC) was analysed separately using a closely related reference genome to maximise the accuracy and resolution of SNP identification. The number of SNP differences between isolates within an outbreak of foodborne disease is considered to range from 0–12 SNPs [41,42,43,44]. However, there is some variation between studies regarding the classification of strains as closely linked. In the current study the criteria described by Kwong and coworkers [41] was used for estimating the possible genomic relatedness of *L. monocytogenes* isolates (Supplementary Material File S1).

The nine ST-1 isolates, including seven clinical isolates and two food processing environment isolates, were compared first using F2365 (ST-1) as the reference genome. Similar numbers of SNPs were revealed when using either of the food processing isolates, L970 or L2113, as the reference genome. Analysing the ST-1 isolates grouped by year of isolation, 2013 or 2014, revealed a relatively high minimum of 44 and 66 SNPs, respectively (Supplementary File S1). The ST-6 and ST-54 isolates also had a high minimum number of SNPs, 199 and 65, respectively (Supplementary File S1). The ST-7 isolates, MQ140029 (clinical) and L1445 (food) were shown to differ by only one SNP (Supplementary File S1) and therefore are phylogenetically highly similar strains [41]. The likelihood of uncovering epidemiological links in historic, sporadic cases of listeriosis is extremely low and indeed we could determine no clear links between this case and the food in question. However, the high phylogenetic similarity between these food and clinical strains indicates that *L. monocytogenes* strains causing cases of clinical infection can be found in Irish foods and highlights the importance of ongoing vigilance to ensure RTE foods remain free of the pathogen.

In our study, the mother–infant ST-121 isolates (one from the mother and one from the infant), MQ140034 and MQ140035, were expected to be highly related and indeed were found to differ by only three SNPs (Supplementary File S1). Similarly, the ST-101 mother–infant isolates (MQ140011 and MQ140012) were found to differ by just three SNPs (Supplementary File S1). The unbiased identification of phylogenetic similarities within each mother–infant pair indicate the validity of the SNP approach for identifying highly related strains.

Finally, the ST-431 isolates, MQ150007 and MQ150008, were found to differ by just two SNPs (Supplementary File S1), indicating that these isolates are likely to be phylogenetically linked [41]. These strains were isolated from separate patients but were isolated by the same local health authority within a one-month period. However, following further retrospective investigation, no epidemiological links between the cases could be determined (personal communication with relevant health authorities). However, the study indicates the ability of SNP analysis to reveal similarities between isolates and to identify the potential for common source outbreaks should they arise. Findings revealed through fine SNP analysis are summarized in Table 2.

### 3.4. Pan- and Core-Genome Analysis

**Figure 3:**
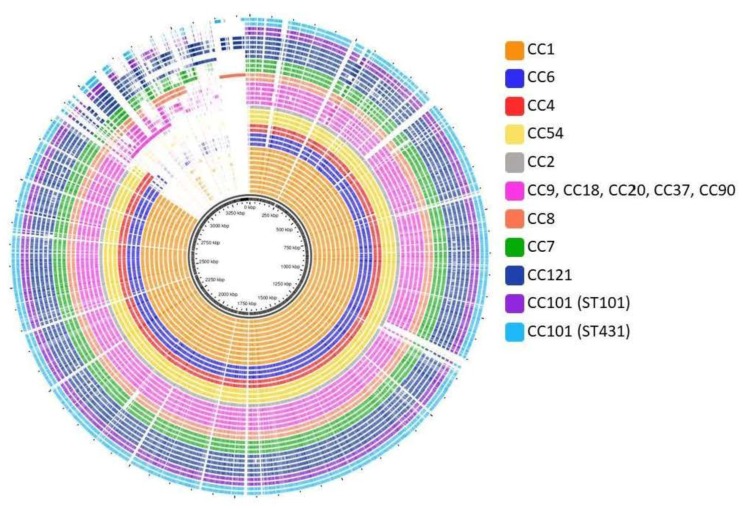
Pan-genome, constructed using GView [35], of 34 genomes including 25 clinical isolates, six food-associated isolates and three reference genomes. The pangenome is constructed by iteratively appending unique regions onto the initial seed genome in this case F2365. Gaps indicate that the region is missing in a particular gene but is found in others. Strains are grouped according to CC and colour coded as indicated.

The pan-genome for 38 isolates, including 25 clinical isolates, six food/food processing environment isolates and seven reference genomes, was generated using GView [35]. Approximately 85% of the coding sequences consist of mutually conserved core genes as shown in Figure 3. This analysis demonstrates that a significant proportion of the *L. monocytogenes* genome is conserved between serotypes as well as STs. Analysis of the accessory genes that are common to a particular CC may yield candidate genes that contribute to successful adaption of isolates to a particular environment [7]. In particular, the significant variation in the accessory genome of ST-121 isolates, indicative of clonal diversity within this group, was noted (Figure 3).

**Figure 4:**
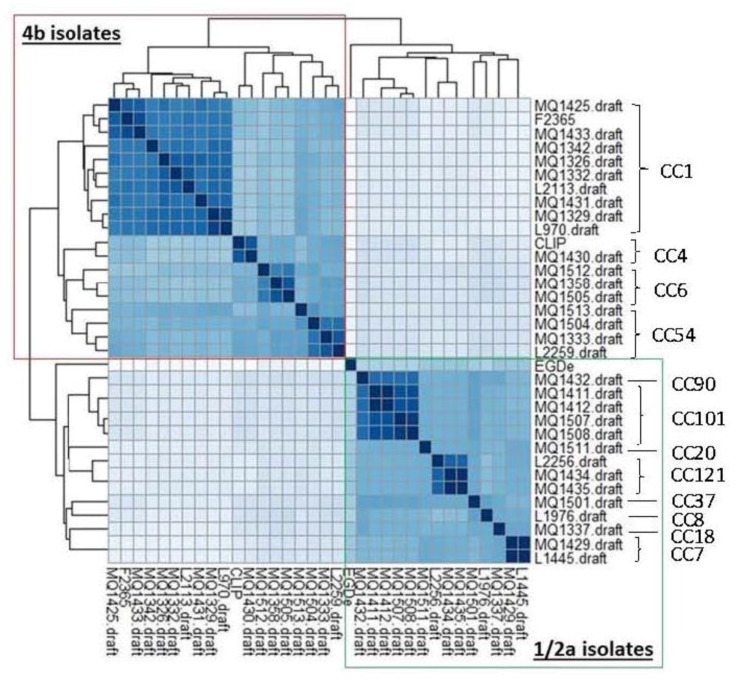
The presence and absence of genes can be used to cluster the isolates into serotypes and sequence types.

The pan-genome of a species is the sum of non-redundant genomic regions from its representative genomes [45]. It is composed of core genomic regions present in all genomes and accessory regions found in some but not all genomes. The *L. monocytogenes* pan-genome has been estimated in several independent studies [45,46,47,48] and has a repertoire of approximately 4000 unique genes. Those studies estimate that there are an average of 2900 genes per strain and approximately 2400 of these genes are part of the core genome (≈80%). A pan-genomic database of orthogolous genes was constructed using PanCoreGen [36]. A cut-off of 80% for sequence identity and gene length coverage resulted in a total of 3105 orthologous genes amongst the genome sequences analysed. The observed core genome shared by the three *L. monocytogenes* reference strains (F2365, CLIP 80459 and EGD-e) is 2431 genes, which is in agreement with the current estimates. These three reference strains cover a minimum of 91% of the coding sequences detected in the draft genomes of the isolates. A matrix of the genetic content of each isolate was generated to indicate the presence and absence of each core and accessory gene. Subsequently, the presence or absence of genes can be used to cluster the isolates into serotypes and CCs (Figure 4).

When implemented as a health-care surveillance approach, the application of systematic WGS of all *L. monocytogenes* isolates, as applied in many different countries recently, can potentially link seemingly sporadic cases of listeriosis to common-source outbreaks [49]. However, although there may be similarities in WGS between strains, linking *L. monocytogenes* isolates to listeriosis outbreaks in the absence of epidemiological data is not feasible.

### 3.5. Listeria Pathogenicity Islands

As indicated, WGS can act as an epidemiological tool to identify highly-related isolates, but it can also be used to identify the presence of genes/pathogenicity islands associated with hypervirulence or particular modes of pathogenesis (such as ability to invade the CNS) [7]. In our study, each of the 31 genomes encodes LIPI-1 which is a Prf-A dependent virulence gene cluster consisting of six genes (*prfA, plcA, hly, mpl*, *actA* and *plcB)* that are key for the infection cycle of *L. monocytogenes* (Table 3). LIPI-3 is a gene cluster that encodes a potential haemolytic factor with homology to Streptolysin S (SLS) [17] and which has recently been shown to possess antimicrobial potential and to play a role in gastrointestinal colonization [18]. LIPI3 is strongly associated with lineage I strains and was found to be present in all Irish serotype 4b isolates with the exception of MQ150013. LIPI-4 was recently described as a gene cluster involved in neural and placental infection [7]. This pathogenicity island encodes six genes annotated as a cellobiose family PTS system and appears to be strongly associated with CC4 isolates. The presence of LIPI4 was confirmed in the only CC4 clinical isolate (MQ140030) in this dataset, a strain originally isolated from the CSF of a patient presenting with listeriosis in 2014. In support of a previous study [7], this island appears to be associated with CC4 and was not identified in any other strains in this study.

### 3.6. Internalins

*L. monocytogenes* encode surface proteins known as internalins that are used to invade host cells [2]. The number of internalins encoded by the genomes ranged from 10 to 13. All isolates encoded full length internalin A with the exception of the food processing isolate L2256. The CC6 isolates (MQ130058, MQ150005 and MQ150012) all have a characteristic three amino acid deletion in the pre-anchor region of InlA [50]. Internalins B, C, C2 (*inlH* in EGDe), E, I and J are also present in all isolates. *inlD* is present in all isolates with the exception of EGDe while *inlF* is present in all isolates with the exception of MQ140034, MQ140035 and L2256.

### 3.7. Stress Survival Islet (SSI-1)

SSI-1 is a five gene islet that contributes to the growth of *L. monocytogenes* under suboptimal conditions [20]. This islet is present in two clinical isolates MQ130037 (CC18), MQ140029 (CC7), and two food-associated isolates L1445 (CC7) and L1976 (CC8). These isolates are all lineage II serotype 1/2. This islet has been recently shown to be a feature of ST-7 (CC7) and ST-8 (CC8) strains associated with persistence in a study of *L. monocytogenes* strains isolated over 20 years from food-processing plants but is also found in sporadic environmental strains [51]. Given that the islet is rare amongst clinical isolates, in this study, it is likely to be dispensable for virulence but may play a role in environmental survival and survival of some strains in foods [20].

## 4. Conclusions

Whole genome sequencing of *L. monocytogenes* isolates from cases of human listeriosis in Ireland between 2013 and 2015 has provided an overview of locally circulating clinical strains of the pathogen. The work identified particular CCs that were responsible for disease in Ireland and permitted comparison of clinical isolates to a comprehensive database of food and food production isolates from the same time period (based upon PFGE type) [21]. SNP analysis revealed that pairs of strains isolated from mother–infant cases were highly related. SNP analysis also identified phylogenetically identical isolates from a patient and a food source and another pair of isolates from distinct patients. Whilst retrospective follow up failed to prove any clear epidemiological links, the study highlights the potential for WGS to identify listeriosis disease patterns within a single national health authority.

## Figures and Tables

**Table 1:** *Listeria monocytogenes* strains sequenced in this study.

Isolate	Genbank Accession Number	ST ^1^	CC ^2^	Lineage	Serotype	Year of Isolation	Sample Type	Pulsotype
MQ130026	MUZG00000000	ST-1	CC1	I	4b	2013	Blood	P2 *
L970	PJJD00000000	ST-1	CC1	I	4b	2013	Food production **	P2 *
MQ130029	MVED00000000	ST-1	CC1	I	4b	2013	CSF ^3^	P2 *
MQ130032	MVEE00000000	ST-1	CC1	I	4b	2013	Blood	P2 *
MQ130042	MVEG00000000	ST-1	CC1	I	4b	2013	Pleural Swab	P1 *
MQ140025	MVEK00000000	ST-1	CC1	I	4b	2014	Ear Swab	P68
MQ140031	MVEN00000000	ST-1	CC1	I	4b	2014	Blood	P2 *
MQ140033	MVEP00000000	ST-1	CC1	I	4b	2014	Blood	P1 *
L2113	PJJE00000000	ST-1	CC1	I	4b	2015	Food production	P2 *
MQ150012	MVEY00000000	ST-6	CC6	I	4b	2015	Blood	P13 *
MQ150005	MVEU00000000	ST-6	CC6	I	4b	2015	Blood	P13 *
MQ130058	MVEH00000000	ST-6	CC6	I	4b	2013	Blood	P13 *
MQ140030	MVEM00000000	ST-4	CC4	I	4b	2014	CSF ^3^	NC ^4^
MQ150004	MVET00000000	ST-54	CC54	I	4b	2015	Placental Swab	P6 *
MQ130033	MVEF00000000	ST-54	CC54	I	4b	2013	Blood	P12
L2259	PJJF00000000	ST-54	CC54	I	4b	2015	Food production	P6 *
MQ150013	MVEZ00000000	ST-2	CC2	I	4b	2015	Blood	P16 *
MQ130037	MVFA00000000	ST-18	CC18	II	1/2a	2013	Blood	P32 *
MQ150011	MVEX00000000	ST-20	CC20	II	1/2a	2015	Nasal Swab	NC
MQ150001	MVES00000000	ST-37	CC37	II	1/2a	2015	Blood	P32 *
MQ140029	MVEL00000000	ST-7	CC7	II	1/2a	2014	Blood	P31 *
L1445	PJJG00000000	ST-7	CC7	II	1/2a	2014	Food production	P31 *
L1976	PJJI00000000	ST-8	CC8	II	1/2c	2015	Food production	P48 *
MQ140034	MVEQ00000000	ST-121	CC121	II	1/2a	2014	Blood—Mother/Infant	P59 *
L2256	PJJH00000000	ST-121	CC121	II	1/2c	2015	Food production	P59 *
MQ140035	MVER00000000	ST-121	CC121	II	1/2a	2014	Ear Swab—Mother/Infant	P59 *
MQ140032	MVEO00000000	ST-425	CC90	II	1/2a	2014	Blood	NC
MQ140012	MVEJ00000000	ST-101	CC101	II	1/2a	2014	Blood—Mother/Infant	P30
MQ140011	MVEI00000000	ST-101	CC101	II	1/2a	2014	Placental Surface Swab—Mother/Infant	P30
MQ150008	MVEW00000000	ST-431	CC101	II	1/2a	2015	Blood	P30
MQ150007	MVEV00000000	ST-431	CC101	II	1/2a	2015	CSF ^3^	P30

^1^ Sequence Type; ^2^ Clonal Complex; ^3^ Cerebrospinal fluid; ^4^ NC = not classified; * Indicates pulsotypes associated with persistence in the food processing environment in a 3 year study of Irish foods and food production facilities [21]; ** These samples are from food or food production environments (non-clinical) and were chosen for sequencing based upon pulsed-field gel electrophoresis (PFGE) similarities to clinical isolates (see text).

**Table 2:** Relatedness of strains as determined by fine single nucleotide polymorphism (SNP) analysis with appropriate reference strains.

Sequence Type	Isolates	Reference Genome	Minimum SNPs	Maximum SNPs
ST1	L970, L2113, MQ130026, MQ130029, MQ130032 *, MQ130042, MQ140025, MQ140031, MQ140033	F2365	43	261
	F2365, L2113, MQ130026, MQ130029, MQ130032 *, MQ130042, MQ140025, MQ140031, MQ140033	L970	42	256
	F2365, L970, MQ130026, MQ130029, MQ130032 *, MQ130042, MQ140025, MQ140031, MQ140033	L2113	42	254
	F2365, L970, MQ130029, MQ130032 *, MQ130042	MQ130026	44	190
	F2365, L2113, MQ140031, MQ140033 *	MQ140025	66	259
ST6	H7858, MQ130058, MQ150012 *	MQ150005	199	373
ST54	LM07-01337, MQ130033, MQ150004 *	L2259	65	115
ST7	J2692, L1846, L2676, MQ140029 *	L1445	1	409
ST121	4423, 6179, L2256, La111, Lm1880, N53-1, MQ140035 *	MQ140034	3	461
ST101	2012-L5240, 2012-L5323, Lm1840, MQ140012, MQ150007, MQ150008 *	MQ140011	1	145
	2012-L5240, 2012-L5323, Lm1840, MQ150008 *	MQ150007	2	146

* Indicates strain with closest similarity to the reference genome (lowest number of SNPs).

**Table 3:** Presence or absence of key loci encoding *L. monocytogenes* virulence-associated elements or loci putatively involved in environmental survival.

Isolate	ST ^1^	CC ^2^	LIPI1	LIPI3	LIPI4	InlA	SSI-1
MQ130026	ST-1	CC1	+ ^3^	+	− ^4^	+ ^5^	−
L970	ST-1	CC1	+	+	−	+	−
MQ130029	ST-1	CC1	+	+	−	+	−
MQ130032	ST-1	CC1	+	+	−	+	−
MQ130042	ST-1	CC1	+	+	−	+	−
MQ140025	ST-1	CC1	+	+	−	+	−
MQ140031	ST-1	CC1	+	+	−	+	−
MQ140033	ST-1	CC1	+	+	−	+	−
L2113	ST-1	CC1	+	+	−	+	−
MQ150012	ST-6	CC6	+	+	−	+	−
MQ150005	ST-6	CC6	+	+	−	+	−
MQ130058	ST-6	CC6	+	+	−	+	−
MQ140030	ST-4	CC4	+	+	+	+	−
MQ150004	ST-54	CC54	+	+	−	+	−
MQ130033	ST-54	CC54	+	+	−	+	−
L2259	ST-54	CC54	+	+	−	+	−
MQ150013	ST-2	CC2	+	−	−	+	−
MQ130037	ST-18	CC18	+	−	−	+	+
MQ150011	ST-20	CC20	+	−	−	+	−
MQ150001	ST-37	CC37	+	−	−	+	−
MQ140029	ST-7	CC7	+	−	−	+	+
L1445	ST-7	CC7	+	−	−	+	+
L1976	ST-8	CC8	+	−	−	+	+
MQ140034	ST-121	CC121	+	−	−	+	−
L2256	ST-121	CC121	+	−	−	−	−
MQ140035	ST-121	CC121	+	−	−	+	−
MQ140032	ST-425	CC90	+	−	−	+	−
MQ140012	ST-101	CC101	+	−	−	+	−
MQ140011	ST-101	CC101	+	−	−	+	−
MQ150008	ST-431	CC101	+	−	−	+	−
MQ150007	ST-431	CC101	+	−	−	+	−

^1^ Sequence Type; ^2^ Clonal Complex; ^3^ Presence of genes; ^4^ Absence of genes; ^5^ Indicates encoding predicted full-length InlA.

## Supplementary Materials

The following are available online at www.mdpi.com/2073-4425/9/3/171/s1, File S1: Fine single nucleotide polymorphism analysis of clinical isolates, Table S1: Number of single nucleotide polymorphisms identified when serotype 4b strains were compared, using F2365 as a reference genome, Table S2: Number of single nucleotide polymorphisms identified when serotype 1/2a strains were compared, using EGDe as a reference genome.
