# Supplementary material for: Genomic Characterization of Listeria monocytogenes Isolates Associated with Clinical Listeriosis and the Food Production Environment in Ireland
Source: Genes (Basel). 2018 Mar 20;9(3):171. doi: 10.3390/genes9030171 (PMC5867892; doi:10.3390/genes9030171)
Supplement: Supplementary file 1 [file genes-09-00171-s001.zip › genes-260027-supplementary for proofs [Corrected].pdf]

genes

MDPI

Supplementary Materials for Genomic Characterization of *Listeria monocytogenes* Isolates Associated with Clinical Listeriosis and the Food Production Environment in Ireland

Amber Hilliard, Dara Leong, Amy O'Callaghan, Eamonn P. Culligan, Ciara A. Morgan, Niall DeLappe, Colin Hill, Kieran Jordan, Martin Cormican and Cormac G.M. Gahan

Supplementary Table S1 Number of single nucleotide polymorphisms identified when serotype 4b strains were compared, using F2365 as a reference genome.

Isolate	L2113	L2259	L970	130026	130029	130032	130033	130042	130058	140025	140030	140031	140033	150004	150005	150012	150013	F2365 *
L2113	0	5434	56	50	56	43	5428	175	5406	247	5392	57	58	5429	5417	5405	5091	162
L2259	5434	0	5440	5434	5442	5431	101	5437	5780	5505	5312	5441	5442	60	5795	5783	5509	5418
L970	56	5440	0	56	62	51	5434	185	5412	251	5398	61	62	5435	5423	5413	5097	170
MQ130026	50	5434	56	0	46	41	5428	177	5406	244	5392	53	48	5429	5417	5405	5091	162
MQ132009	56	5442	62	46	0	47	5436	185	5414	253	5400	59	54	5437	5425	5415	5099	170
MQ130032	43	5431	51	41	47	0	5425	172	5403	242	5389	48	49	5426	5414	5404	5088	159
MQ130033	5428	101	5434	5428	5436	5425	0	5431	5771	5499	5305	5435	5436	95	5786	5774	5501	5412
MQ130042	175	5437	185	177	185	172	5431	0	5409	250	5395	186	187	5432	5420	5410	5092	137
MQ130058	5406	5780	5412	5406	5414	5403	5771	5409	0	5477	5849	5413	5414	5773	91	77	5587	5388
MQ140025	247	5505	251	244	253	242	5499	250	5477	0	5463	252	253	5500	5488	5478	5162	233
MQ140030	5392	5312	5398	5392	5400	5389	5305	5395	5849	5463	0	5399	5400	5306	5862	5850	5665	5372
MQ140031	57	5441	61	53	59	48	5435	186	5413	252	5399	0	59	5436	5424	5414	5098	171
MQ140033	58	5442	62	48	54	49	5436	187	5414	253	5400	59	0	5437	5425	5415	5099	172
MQ150004	5429	60	5435	5429	5437	5426	95	5432	5773	5500	5306	5436	5437	0	5788	5776	5502	5413
MQ150005	5417	5795	5423	5417	5425	5414	5786	5420	91	5488	5862	5424	5425	5788	0	100	5605	5397
MQ150012	5405	5783	5413	5405	5415	5404	5774	5410	77	5478	5850	5414	5415	5776	100	0	5590	5389
MQ150013	5091	5509	5097	5091	5099	5088	5501	5092	5587	5162	5665	5098	5099	5502	5605	5590	0	5069
F2365 *	162	5418	170	162	170	159	5412	137	5388	233	5372	171	172	5413	5397	5389	5069	0
min: 41 max: 5862																		

* Reference strain

Supplementary Table S2. Number of single nucleotide polymorphisms identified when serotype 1/2a strains were compared, using EGDe as a reference genome.

Isolate	L1445	L1976	L2256	130037	140011	140012	140029	140032	140034	140035	150001	150007	150008	150011	EGDe *
L1445	0	10146	12511	10287	11637	11636	6	11825	12482	12479	10236	11631	11641	10094	9522
L1976	10146	0	12230	9814	11569	11568	10150	11656	12197	12194	9312	11565	11571	9701	8263
L2256	12511	12230	0	12182	11203	11202	12515	11068	173	170	12273	11193	11203	12259	11363
MQ130037	10287	9814	12182	0	11557	11556	10291	11560	12145	12142	9623	11549	11555	9618	7926
MQ140011	11637	11569	11203	11557	0	3	11641	4534	11168	11165	11656	97	85	11589	10668
MQ140012	11636	11568	11202	11556	3	0	11640	4533	11167	11164	11655	96	84	11588	10667
MQ140029	6	10150	12515	10291	11641	11640	0	11829	12486	12483	10240	11635	11645	10096	9526
MQ140032	11825	11656	11068	11560	4534	4533	11829	0	11037	11034	11617	4530	4534	11720	10683
MQ140034	12482	12197	173	12145	11168	11167	12486	11037	0	3	12244	11164	11168	12226	11330
MQ140035	12479	12194	170	12142	11165	11164	12483	11034	3	0	12241	11161	11165	12223	11327
MQ150001	10236	9312	12273	9623	11656	11655	10240	11617	12244	12241	0	11648	11658	9545	8589
MQ150007	11631	11565	11193	11549	97	96	11635	4530	11164	11161	11648	0	12	11581	10663
MQ150008	11641	11571	11203	11555	85	84	11645	4534	11168	11165	11658	12	0	11591	10673
MQ150011	10094	9701	12259	9618	11589	11588	10096	11720	12226	12223	9545	11581	11591	0	8980
EGDe *	9522	8263	11363	7926	10668	10667	9526	10683	11330	11327	8589	10663	10673	8980	0
min: 3 max: 12515															

* Reference strain
